# Identification of Single Nucleotide Non-coding Driver Mutations in Cancer

**DOI:** 10.3389/fgene.2018.00016

**Published:** 2018-02-02

**Authors:** Kok A. Gan, Sebastian Carrasco Pro, Jared A. Sewell, Juan I. Fuxman Bass

**Affiliations:** Department of Biology, Boston University, Boston, MA, United States

**Keywords:** cancer, non-coding mutation, driver mutation, hotspot analysis, motif analysis

## Abstract

Recent whole-genome sequencing studies have identified millions of somatic variants present in tumor samples. Most of these variants reside in non-coding regions of the genome potentially affecting transcriptional and post-transcriptional gene regulation. Although a few hallmark examples of driver mutations in non-coding regions have been reported, the functional role of the vast majority of somatic non-coding variants remains to be determined. This is because the few driver variants in each sample must be distinguished from the thousands of passenger variants and because the logic of regulatory element function has not yet been fully elucidated. Thus, variants prioritized based on mutational burden and location within regulatory elements need to be validated experimentally. This is generally achieved by combining assays that measure physical binding, such as chromatin immunoprecipitation, with those that determine regulatory activity, such as luciferase reporter assays. Here, we present an overview of *in silico* approaches used to prioritize somatic non-coding variants and the experimental methods used for functional validation and characterization.

## Introduction

Cancer initiation, progression, maintenance, and metastasis originate from somatic single nucleotide variants (SNVs), small insertions and deletions, structural variants, and epigenetic alterations (Helleday et al., 2014). In particular, recent whole-genome sequencing studies of tumor samples, through collaborative projects such as The Cancer Genome Atlas (TCGA) and the International Cancer Genome Consortium (ICGC), have identified millions of somatic SNVs associated with different types of cancers (Cancer Genome Atlas Research Network, 2008, 2013; Nik-Zainal et al., 2016). Although, these projects and follow-up studies have been successful at identifying common sets of mutated genes and pathways across many cancer types, the functional role of most mutations detected remains to be determined. Indeed, the main challenge in analyzing the genetics underlying cancer is to distinguish driver mutations (i.e., positively selected mutations that provide growth advantage to tumor cells) from passenger mutations (i.e., inert mutations that do not confer any growth advantages) (Khurana et al., 2016). This requires the integration of computational analyses that predict functional SNVs with experimental pipelines to validate and characterize those SNVs.

**FIGURE 1:**
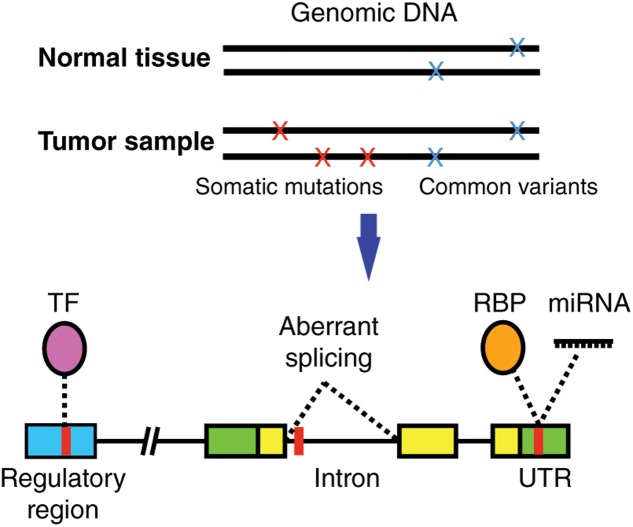
Non-coding cancer mutations affecting transcriptional and post-transcriptional regulation. Somatic mutations (present in tumor but not in matched normal tissue samples) can alter gene regulation by affecting the binding of a transcription factor (TF) to a regulatory region, the binding of RNA binding proteins (RBPs) or miRNAs to untranslated regions (UTRs) in the mRNAs, or affect normal splicing. TF, purple; RBP, orange; regulatory region, blue; UTR, green; coding region, yellow; SNV, red.

Most studies have focused on characterizing the functional impact of SNVs on coding regions given that it is relatively straightforward to computationally predict how a protein sequence and/or structure will be affected by a missense, nonsense or frameshift mutation. However, the vast majority of SNVs identified in cancer samples reside in non-coding regions of the genome (Araya et al., 2016). These non-coding SNVs can affect the binding of transcription factors (TFs), RNA-binding proteins (RBPs), and micro RNAs (miRNAs) (**Figure 1**) (Khurana et al., 2016). This in turn affects multiple gene regulatory functions including chromatin structure or accessibility, transcription, DNA methylation, splicing, as well as 5′ and 3′ untranslated region (UTR) function, which ultimately increases or decreases the production, stability and translation efficiency of mRNA transcripts (Khurana et al., 2016).

Despite recent advances in the understanding of the downstream consequences of non-coding SNVs, it remains a challenge to identify non-coding driver mutations and the mechanisms through which they effect biological functions. First, as stated above, non-coding SNVs can affect multiple regulatory functions including transcriptional and post-transcriptional regulation. Second, non-coding regions present higher mutations rates than coding regions, due to weaker selective pressure (Weinhold et al., 2014). As a result, parsing through a higher number of passenger mutations to find non-coding driver SNVs becomes a difficult statistical and computational task (Vogelstein et al., 2013). Third, it is challenging to computationally predict whether a non-coding SNV affects gene expression or mRNA stability because the logic involved in regulatory element function has not yet been fully elucidated. Thus, computational predictions of altered regulatory function need to be confirmed by extensive experimental validation using reporter assays, genome editing, measurement of endogenous gene expression, and/or chromatin immunoprecipitation.

Early studies that identified non-coding driver SNVs compared the sequence of regulatory regions of candidate cancer-related genes between tumor and non-tumor samples in order to determine whether these mutations disrupt or create TF binding sites. For example, SNVs were identified in the GTAAC sequence within the first intron of MYC in samples from multiple patients with Burkitt lymphomas (Zajac-Kaye et al., 1988). These mutations, which lead to increased MYC expression, abrogated the binding of a then unidentified TF. Since this early work, targeted studies have identified several mutations in regulatory regions, both in tumor samples and in patients with increased cancer incidence (Stenson et al., 2009).

More recently, whole-genome sequencing of matched tumor and normal samples has enabled the identification of millions of SNVs. However, the identity of the SNVs responsible for driving cancer and those that constitute passenger mutations remains to be determined. Two pioneering studies showed that mutations present in the telomerase reverse transcriptase (TERT) promoter in tumor samples of patients with melanoma lead to increased TERT mRNA expression (Horn et al., 2013; Huang et al., 2013). These studies identified two independent C > T transitions, at around -100 bp from the TERT transcription starting site (TSS), that create a 11 bp nucleotide stretch containing a consensus binding site for E-twenty-six (ETS) TFs. Additionally, other mutations in the TERT promoter have been found in melanoma as well as in other cancer types such as ovarian, follicular thyroid, and meningiomas (Horn et al., 2013; Goutagny et al., 2014; Liu et al., 2014; Wu et al., 2014). More recently, mutations in the regulatory regions of other cancer-related genes have been identified, including recurrent mutations in the promoters of PLEKHS1, WDR74, SDHD, and FOXA1 that alter gene expression levels, TF binding and that are associated with poor prognosis (Fredriksson et al., 2014; Weinhold et al., 2014; Nik-Zainal et al., 2016; Rheinbay et al., 2017). Here, we present an overview of state-of-the-art approaches to computationally predict and functionally validate driver somatic non-coding SNVs, as well as recent findings associated with cancer.

## Computational Approaches to Identify Non-Coding SNVs

**FIGURE 2:**
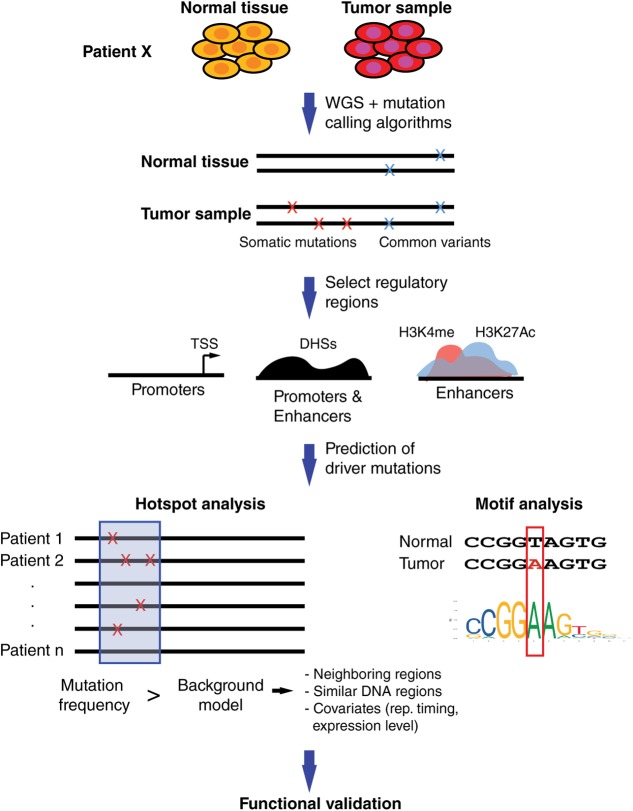
Computational pipeline to prioritize somatic SNVs in regulatory elements. Whole-genome sequencing (WGS) of matched tumor and normal samples are analyzed to identify somatic mutations. Identification of mutations within regulatory regions is performed by restricting analyses to promoter regions, generally defined around transcription start sites, and distal elements such as enhancers, predicted based on DHSs and/or histone marks. Hotspot analyses are used to identify regions with increased mutational burden compared to background models based on mutational frequency in neighboring regions and/or regions with similar functional roles. Covariates such as replication timing or gene expression levels can be included to account for mutational heterogeneity across the genome. Motif analyses are performed to predict differential TF binding between SNV alleles. Prioritized non-coding SNVs are usually validated in functional assays.

Computational approaches to predict functional SNVs within regulatory regions share a common general pipeline, including the identification of somatic SNVs, comparison with common germline variants, constraining the analysis to regulatory regions (in some cases, close to cancer-related genes), identification of mutational hotspots, and determining altered TF binding sites (**Figure 2**).

The identification of somatic SNVs requires comparing the genome sequences of tumor samples with matched normal tissue samples. This is a challenging task because somatic SNVs occur at low frequency in the genome (0.1–100 SNVs per megabase), which needs to be distinguished from errors derived from whole-genome sequencing and genome alignment pipelines (Lawrence et al., 2013; Alioto et al., 2015). Thus, most methods used to identify somatic SNVs require high sequencing depths (usually 30–300x) and paired-end reads, leading to elevated sequencing costs (Alioto et al., 2015). In addition, given that tumors are comprised of heterogeneous populations of cells, many functional SNVs may be present at a low frequency in patient samples (Carter et al., 2012; Nik-Zainal et al., 2012b). Therefore, while high-frequency SNVs can be identified provided that the sequencing depth is sufficient enough and that computational pipelines accommodate for sequence heterogeneity, low-frequency SNVs may require single-cell genome sequencing approaches (Navin et al., 2011; Zong et al., 2012; Eirew et al., 2015).

Several computational methods have been developed to identify somatic SNVs, including: (1) those that separately call SNVs in tumor and normal samples and then identify tumor-specific SNVs by comparison, such as GATK (DePristo et al., 2011), GATKcan (Hsu et al., 2017), and EBCall (Shiraishi et al., 2013); and (2) those that concurrently analyze tumor-normal samples using heuristic methods or statistical models, such as MuTect (Cibulskis et al., 2013), VarScan (Koboldt et al., 2009, 2012), and Strelka (Saunders et al., 2012) (**Table 1**). While the first type of methods models sequencing errors based on statistical parameters from the sequencing reads or from non-matched normal samples, the second type of methods compare matched tumor-normal samples to distinguish true mutations from sequencing errors. Even though these algorithms have been used as stand-alone methods to call SNVs, some studies have used a combination of methods for a “wisdom of the crowd” approach with the goal of increasing the confidence in the SNVs detected (Weinhold et al., 2014; Melton et al., 2015).

**Table 1:** List of computational methods and databases to identify somatic SNVs, incorporate background models to predict functional non-coding SNVs, predict altered TF binding sites, and integrate with functional annotations.

Goal	Method/database	Reference
	GATK	DePristo et al., 2011
	GATKcan	Hsu et al., 2017
Identification of somatic	EBCall	Shiraishi et al., 2013
SNVs	MuTect	Cibulskis et al., 2013
	Varscan	Koboldt et al., 2009
	Varscan2	Koboldt et al., 2012
	Strelka	Saunders et al., 2012

Incorporation of	MutSigNC	Rheinbay et al., 2017
background models for	LARVA	Lochovsky et al., 2015
non-coding SNVs	MOAT	Lochovsky et al., 2017

	FIMO	Grant et al., 2011
	MotifbreakR	Coetzee et al., 2015
	BEEML-PBM	Hume et al., 2015
	TFM-pvalue	Touzet and Varre, 2007
Prediction of TF	MotifLocator	Claeys et al., 2012
binding sites	CIS-BP	Weirauch et al., 2014
	Jaspar	Khan et al., 2017
	Uniprobe	Hume et al., 2015
	Transfac	Matys et al., 2003

	RegulomeDB	Boyle et al., 2012
Integration with	Funseq2	Fu et al., 2014
functional annotation of non-coding regions	ENCODE Project	ENCODE Project Consortium, 2012
	Roadmap Epigenomics	Roadmap Epigenomics Consortium et al., 2015
	FANTOM Consortium	Andersson et al., 2014
	GTEx Project	GTEx Consortium, 2013

*This list is not exhaustive, thus, the authors apologize for any method/database not referenced in this table*.

## Hotspot Analyses Based on Mutation Frequency

Among the millions of non-coding somatic SNVs identified in different cancers, only a small number are expected to be drivers. Given that it is not currently possible to experimentally test most of the SNVs identified, methods have been developed to prioritize which SNVs are more likely to be functional. A common approach to prioritize somatic SNVs is to determine genomic regions with high mutation frequency across different cancer samples. Given the billions of bases in the human genome, the thousands of mutations per cancer sample, and that we only have sequencing data for a few thousand tumors, the chances of detecting a significantly enriched mutation across cancers after multiple hypothesis testing correction is almost null.

Currently, there are two complementary strategies, frequently used together, to increase the power to detect non-coding driver mutations. One strategy is to focus on DNA elements that are expected to have a regulatory function. For example, promoter regions are relatively easy to determine by selecting regions up- and downstream of transcription start sites, while distal elements are usually determined based on DNase hypersensitivity sites (DHSs) or histone marks such as H3K4me and H4K27ac (**Figure 2**) (ENCODE Project Consortium, 2012). Further, some studies constrain the analyses to the regulatory regions of cancer-related genes such as those compiled in the Cancer Gene Census (Futreal et al., 2004). Overall, restricting the analysis to a set of regulatory regions reduces the search space for SNVs and, thus increases the power to detect driver mutations.

The second strategy is the identification of clusters of SNVs within short DNA windows, called hotspots, rather than single mutations (**Figure 2**). This reduces dimensionality and increases the frequency of SNVs within each DNA window leading to increased statistical power. The identification of these mutational hotspots across cancers involves comparing the SNV frequency within a DNA window to a background distribution of SNV frequencies. These methods can be divided into local and global models, comparing the SNV frequencies to other windows in neighboring genomic regions or to functionally similar regions (e.g., other promoters or enhancers), respectively. The window size selection can vary widely between analysis, ranging from 50 bp (Weinhold et al., 2014) up to 500 kb (Fujimoto et al., 2016). While short windows provide higher resolution, allowing one to identify functional promoter or enhancer regions, they lead to low statistical power and thus many functional regions may be missed (Fujimoto et al., 2016). Long windows do not have the resolution to detect functional promoters or enhancers but allow for the identification of covariates, regional features associated with genomic heterogeneity in mutation frequency, such as replication timing and gene expression levels (Fujimoto et al., 2016). Both types of methods can be integrated with one another to increase the chances of detecting driver mutations. For example, a recent study analyzing 863 human tumors has identified recurrent mutations in regulatory elements upstream of TERT, PLEKHS1, WDR74 and SDHD in different types of cancer by using 50 bp windows to find hotspots, and regional recurrence approaches that take into account length and replication timing (Weinhold et al., 2014).

Although studies using low tumor sample numbers may be underpowered to identify hotspot regions, large samples sizes can also be challenging to analyze. This is because large sample sizes frequently lead to larger lists of potentially significant genes which in many cases do not have cancer-related functions, suggestive of a high false positive prediction rate (Lawrence et al., 2013). This stems from using background mutation models that do not account for mutational heterogeneity between samples and across genomic regions (Lawrence et al., 2013). Pipelines such as MutSigNC have been developed to correct for variation in mutation frequency by considering patient-specific mutation rates, patient-specific sequencing coverage, information about regional mutation clustering, and using as background the mutation rates of promoters (Rheinbay et al., 2017) (**Table 1**). Other computational frameworks have also been used to also include distal elements in the analyses, including LARVA that incorporates background models for non-coding regions by integrating SNVs with a comprehensive set of non-coding functional elements based on DHSs and histone marks (Lochovsky et al., 2015) (**Table 1**). In addition, LARVA uses regional genomic features like replication timing allowing to better estimate local mutation rates and mutational hotspots.

Further covariates can be included while modeling mutation frequencies. For instance, recent studies have shown that some breast tumors have mutations mediated by the alipoprotein B messenger RNA-editing enzyme catalytic (APOBEC) which have been found to occur in dense hypermutated regions in the genome (kataegis) (Nik-Zainal et al., 2012a; Alexandrov et al., 2013). These mutations share a sequence pattern (TCW, where W is A/T), which can be used to assign mutations a probability of being originated by APOBEC activity (Roberts et al., 2013), leading to a more conservative approach to call candidate mutations. This approach identified SNVs in breast cancer samples within the regulatory regions of FOXA1, RMRP, and NEAT1 that affect gene expression levels (Rheinbay et al., 2017). Alternatively, covariates can be avoided altogether by using a non-parametric, permutation-based approach such as MOAT, that does not make assumptions about the mutation process except for requiring that the background-mutation rate changes smoothly with genomic features (Lochovsky et al., 2017) (**Table 1**). The variety of co-existing computational approaches, background models, and covariates included in those models, highlights the challenges currently faced in identifying mutational hotspots associated with cancer.

## Prediction of Non-Coding SNVs with High Functional Impact

Hotspot analyses allow for the prioritization of candidate cancer driver SNVs. However, to further narrow down the set of functional SNVs and predict the functional impact of these SNVs, location and sequence context of the mutations must be integrated with functional models of non-coding regions. One of the most widely used approaches to prioritize SNVs in regulatory regions involves the identification of TF binding sites created or disrupted by the mutations (**Figure 2**). These TF binding differences between SNV alleles can be predicted based on DNA specificities determined by protein-binding microarrays, SELEX, bacterial one-hybrid assays, or chromatin immunoprecipitation (ChIP) followed by next generation sequencing (ChIP-seq) (Noyes et al., 2008; Jolma et al., 2013; Weirauch et al., 2014). Currently, DNA binding specificities have been determined for nearly half of human TFs, which are available in different repositories such CIS-BP, Jaspar, Uniprobe, and Transfac (Matys et al., 2003; Weirauch et al., 2014; Hume et al., 2015; Khan et al., 2017) (**Table 1**). Differences in TF binding between SNV alleles can be predicted using position weight matrices (PWMs), probabilistic representations of DNA binding specificities, and motif prediction algorithms such as FIMO (Grant et al., 2011), MotifbreakR (Coetzee et al., 2015), BEEML-PBM (Hume et al., 2015), TFM-pvalue (Touzet and Varre, 2007), and MotifLocator (Aerts et al., 2005; Claeys et al., 2012) (**Table 1**). For example, MotifLocator, a tool to score how mutations affect wild-type TF binding sites, led to the identification of gain of binding sites for RB1, E2F1 and ETS to multiple promoter regions in tumor samples from TCGA (Kalender Atak et al., 2017). Similarly, mutations in the promoter of FOXA1, a known gene driver in breast cancer, were found to increase E2F binding using TFM-pvalue (Rheinbay et al., 2017). Loss of TF binding sites have also been widely associated with cancer. For example, many recurrent mutated regions in cancer genomes have been found to overlap with CTCF binding sites, showing a possible selection for these mutations (Katainen et al., 2015; Lochovsky et al., 2015; Piraino and Furney, 2017). In addition, disruption of FOX TF binding sites in the BCL6 promoter have been reported in follicular lymphoma using an integrative approach that identifies functional regulatory mutation blocks (Batmanov et al., 2017). Interestingly, both the creation and disruption of binding sites for the same TFs have been linked to cancer. For example, by integrating motif analyses with evolutionary conservation, creation of ETS binding sites were determined in the ANKRD53 promoter, while disruption of ETS binding sites were identified in the TAF11 and SDHD promoters (Weinhold et al., 2014).

In addition, motif analyses can integrate functional annotations of regulatory sequences (including DHSs, histone marks, and sequence conservation) and TF expression levels such as those provided by the ENCODE, Roadmap Epigenomics, FANTOM, and GTEx Projects to constrain the analyses to TFs expressed and regulatory elements active in the tissues of interest (ENCODE Project Consortium, 2012; GTEx Consortium, 2013; Andersson et al., 2014; Roadmap Epigenomics Consortium et al., 2015) (**Table 1**). These approaches include RegulomeDB (Boyle et al., 2012) that considers functional annotations for the regulatory regions, and Funseq2 (Fu et al., 2014) that also considers sequence conservation across species and recurrence of somatic mutations in cancer (**Table 1**).

Although motif analyses have been instrumental to predict altered TF binding, these methods are limited by the availability of high-quality PWMs and by the high false positive and false negative predictions rates of motif finding algorithms (Zia and Moses, 2012; Weirauch et al., 2014; Sewell and Fuxman Bass, 2017). Indeed, motif analyses can rarely distinguish between different members of a TF family, and often miss the TF that differentially binds to SNV alleles (Weirauch et al., 2014). Thus, SNVs in regulatory regions predicted to be functional based on hotspot and motif analyses, need to be experimentally tested to determine whether these mutations actually affect TF binding.

## Experimental Validation of Differential TF Binding Between SNV Alleles

**FIGURE 3:**
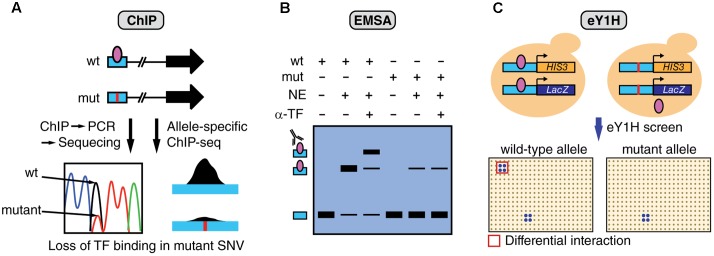
Overview of assays to measure differential TF binding between non-coding SNV alleles. **(A)** ChIP against a candidate TF can be performed in cells that are heterozygous for the SNV. Sequencing of the amplified regions (or allele-specific qPCR) can determine relative TF binding between wild-type (wt) and mutant (mut) alleles. Alternatively, ChIP-seq data can be analyzed to detect biases in the number of sequencing reads between alleles. The figure shows an example of loss of TF binding caused by a mutation. **(B)** EMSA can be performed to determine differential TF binding to oligonucleotides containing wt or mut SNV alleles by using nuclear extracts (NE) followed by super-shifts using antibodies against the candidate TF (α-TF), or by incubating with extracts overexpressing the TF. **(C)** eY1H assays can test the binding of >1,000 TFs to wild-type and mutant allele sequences. In this assay, each DNA sequence is cloned upstream the *HIS3* and *LacZ* reporters and integrated into the yeast genome. Interactions are tested by mating with yeast strains expressing different TFs in an arrayed format system. Differential TF interactions (highlighted in red) can be determined by comparing screening results between alleles.

Multiple complementary experimental methods can be used to determine TF binding including ChIP, electrophoretic mobility shift assays (EMSA), and enhanced yeast one-hybrid (eY1H) assays (**Figure 3**). ChIP has been successfully used to study differential TF binding between non-coding SNV alleles *in vivo* (**Figure 3A**). For example, several studies have identified mutations in the TERT promoter, such as G228A, that lead to the creation of *de novo* bind site for ETS factors (Horn et al., 2013; Huang et al., 2013). However, the identity of the specific ETS factor involved remained elusive until a recent study analyzing ChIP-seq data from the ENCODE Project (ENCODE Project Consortium, 2012), identified GABPA as the TF that differentially binds and regulates TERT expression (Bell et al., 2015). In particular, GABPA was found to be bound to the TERT promoter in heterozygote cell lines harboring the G228A mutation, specifically to the mutant allele, while other ETS factors did not show significant binding. Although ChIP is the method of choice to validate *in vivo* differential TF binding between alleles, this method requires *a priori* TF candidates as it can only test one TF at a time. Further, given that ChIP tests for *in vivo* TF binding, experiments need to be performed in cell lines harboring the mutations or using patient samples, which are frequently challenging to obtain.

A recent study using enhanced yeast one-hybrid (eY1H) assays, a method that tests protein-DNA interactions in the milieu of the yeast nucleus, has increased the screening throughput for TF binding differences between SNV alleles by testing > 1,000 TFs in parallel, without the need for antibodies or patient samples (**Figure 3C**) (Fuxman Bass et al., 2015). Although this study has focused on germline variants associated with different genetic diseases, the experimental eY1H pipeline can also be used to evaluate somatic SNVs in cancer. Given that ChIP, EMSA and eY1H assays measure physical DNA binding, rather than regulatory activity, interactions identified by these methods need to be tested in human cell lines to determine the SNV impact on gene regulation by using transient reporter assays, or endogenous gene expression measurements following TF knockdown/knockout.

## Experimental Validation of Altered Gene Expression by SNVs

**FIGURE 4:**
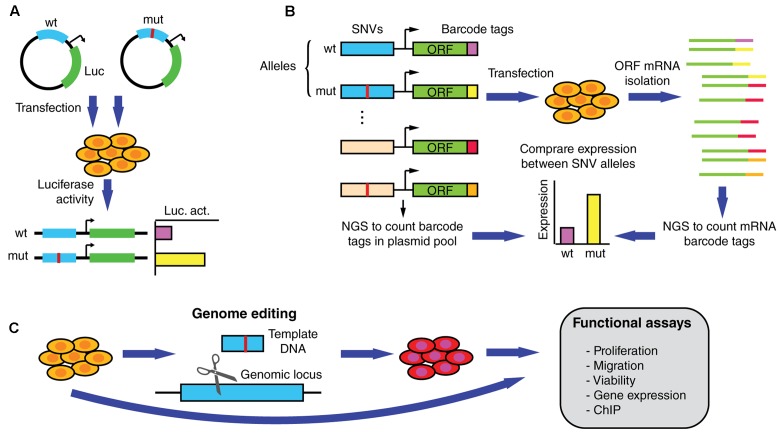
Functional assays to measure altered gene expression and phenotypic parameters induced by SNVs in regulatory regions. **(A)** Reporter assays can be used to determine differential expression induced by wild-type and mutant regulatory elements in transiently transfected cells. **(B)** In MPRAs wild-type and mutant alleles for hundreds/thousands of non-coding SNVs can be tested in parallel for changes in transcriptional activity. ∼200 bp sequences containing the SNVs are cloned upstream of an inert ORF and associated with random barcodes. Cells are then transfected with the pooled library, ORF-specific mRNA is isolated, and barcode tags are counted using next-generation sequencing (NGS). By comparing the number of reads per allele in the mRNA and the plasmid populations, relative expression levels can be determined. **(C)** Functional validation and follow-up studies can be performed by determining differences in endogenous gene expression, proliferation, migration, and viability, among other assays, using cells engineered to carry the mutation.

Driver mutations that affect regulatory regions are expected to affect the expression of a target gene. Functional validation assays such as those using luciferase reporters have been widely used to determine expression differences between non-coding SNV alleles (**Figure 4A**) (Huang et al., 2013; Denisova et al., 2015; Fuxman Bass et al., 2015; Rheinbay et al., 2017). In addition, reporter assays can be used to validate differential TF binding determined based on physical binding assays, by overexpressing or knocking down TF expression and measuring the impact on reporter activity driven by the wild-type or mutant regulatory sequences. Although useful for functional validation, reporter assays are generally low-throughput and cannot keep pace with the discovery of new mutations.

Recent studies using massively parallel reporter assays (MPRAs), a high-throughput technology based on barcodes and next generation sequencing, have made progress in determining whether germline SNVs associated with genetic disorders affect transcriptional regulation (**Figure 4B**) (Melnikov et al., 2012; Mogno et al., 2013; Tewhey et al., 2016; Ulirsch et al., 2016). In particular, differential transcriptional activity has been detected for hundreds of expression quantitative trait loci (eQTL) and disease-associated variants. While this method remains to be applied to cancer SNVs, it is expected that MPRAs will constitute an essential tool for identifying functional non-coding somatic SNVs. Although powerful, MPRAs are not free of caveats. For instance, current oligonucleotide synthesis pipelines only allow for a maximum DNA fragment length of ∼230 nucleotides. Thus, non-coding mutations are not usually tested within full length regulatory elements (that can be up to several kilobases), which may be hamper the ability of MPRAs to detect changes in gene expression. This limitation may be overcomed as pooled and arrayed oligonucleotide synthesis technologies are adapted to generate longer DNA sequences. Another limitation of MPRAs is that reporter activity is generally tested using episomal constructs, or randomly integrated lentiviral constructs, that do not reflect the endogenous genomic context where the non-coding mutations reside (Tewhey et al., 2016; Ulirsch et al., 2016). Thus, the functional effect of many SNVs on target gene expression may be over or underestimated. Downstream validation studies in the appropriate genomic context can be conducted by introducing the SNV in the endogenous locus using genome editing technologies such as the CRISPR/Cas9 system, zinc finger nucleases, or transcription activator-like effector nucleases (**Figure 4C**) (Claussnitzer et al., 2015; Elkon and Agami, 2017). These studies, ultimately need to be followed-up using assays that demonstrate the biological significance of the SNVs in cancer by measuring different oncogenic properties such as invasion, proliferation, and viability (**Figure 4C**).

## SNVs Affecting Distal Regulatory Elements

Compared to promoters, dissecting the functional effects of mutations in distal regulatory elements such as enhancers is a more complex task as it is not trivial to determine which of these elements are functional in different cells/conditions nor the identity of the target gene involved. This, and the fact that including distal elements in hotspot analyses increases the search space and reduces statistical power are the main reasons why most studies characterizing germline and somatic non-coding SNVs have focused on promoter regions (Stenson et al., 2009; Rheinbay et al., 2017).

Several technologies have been used to identify promoter-enhancer pairs interacting through chromatin loops. These methods, that involve crosslinking and ligation of spatially closed genomic regions, such as Hi-C (Lieberman-Aiden et al., 2009) and chromatin conformation capture by paired-end tag sequencing (ChIA-Pet) (Li et al., 2012), have been used to capture the potential regulatory effect of enhancer mutations. For example, a recent study found that a somatic SNV (C > T) four kilobases upstream of the transcriptional start site of the *LMO1* oncogene generated a *de novo* binding site for the MYB TF in patients with T-cell acute lymphoblastic leukemia (Li Z. et al., 2017). A combination of ChIP-Seq of MYB, followed by ChIA-PET and luciferase assays revealed that this mutation induced the formation of an aberrant transcriptional enhancer complex leading to increased expression of the *LMO1* oncogene. Thus, integration of chromatin interaction data can identify the gene targets of distal regulatory elements and determine how mutations in those elements affect looping interactions leading to changes in gene expression.

## Non-Coding SNVs Affecting Post-Transcriptional Regulation

Non-coding mutations not only affect transcriptional regulation but can also affect other biological processes such as mRNA stability, translation efficiency, or splicing. Mutations in UTRs can affect mRNA stability and translation efficiency by altering interactions with RNA-binding proteins and miRNAs (**Figure 1**) (Khurana et al., 2016). For example, mutations in the 5′ UTR of *RB1* alter UTR conformation and mRNA stability in retinoblastoma (Kutchko et al., 2015), while mutations in the 5′ UTR of *BRAC1* in breast cancer patients reduce translation efficiency (Signori et al., 2001; Wang et al., 2007). In addition, mutations in the 3′ UTR of *BRCA1* were found to introduce a functional miRNA-103 target site in a breast cancer case leading to reduced *BRAC1* levels (Brewster et al., 2012). As with SNVs in transcriptional regulatory regions, the functional impact of UTR mutations need to be tested in experimental assays. Low-throughput reporter assays have been used to quantify differences in mRNA levels by cloning the relevant UTR regions upstream or downstream of the coding region of GFP or luciferase. More recently, massively parallel functional annotation of sequences from 3′ UTRs (fast-UTR) has been developed, which was used to discover 87 novel *cis*-regulatory elements and measure the effects of known gene variations in 3′ UTRs (Zhao et al., 2014).

Mutations in the exon–intron boundaries, introns, and coding regions can affect splicing and lead to the upregulation oncogenic isoforms or the downregulation of tumor suppressor isoforms. Various cancer tumor suppressor genes such as TP53, ARID1A, PTEN, CHD1, MLL2, and PTCH1 were found to carry mutations in the exon–intron boundaries which led to intron retention (Supek et al., 2014; Jung et al., 2015). For example, an intronic mutation in *BRAF* induces the expression of a splice variant that confers resistance to vemurafenib treatment in melanoma (Salton et al., 2015). These aberrant or cancer-specific isoforms are generally detected using short- and/or long-read mRNA sequencing, and are usually validated using mini-gene constructs carrying the different SNV alleles in low- or high-throughput assay formats (Gaildrat et al., 2010; Cavelier et al., 2015; Rosenberg et al., 2015; Li Y. et al., 2017).

## Future Perspectives

Recent studies have identified a handful of somatic SNVs in regulatory regions that affect TF binding and target gene expression. However, the number of functional non-coding SNVs associated with cancer is expected to be much higher given the low overlap between those reported in different studies, and given that non-coding SNVs seem to play an important role in disease based on the hundreds of functional non-coding SNVs identified in genome-wide association and genetic studies (Stenson et al., 2009). Advances in several areas will be needed to increase our ability to identify these driver mutations. First, larger numbers of tumor samples with available whole-genome sequence data are needed to increase statistical power in prediction algorithms. Second, more refined background models in hotspot analyses that take into account multiple covariates will help identify functional regulatory regions in cancer. Finally, improvements in motif analyses will be needed through the generation of PWMs for uncharacterized TFs and by identifying *in silico* parameters that can accurately predict differential TF binding between alleles.

Another source of underestimation of non-coding driver SNVs stems from the hotspot analysis itself as it assumes that driver mutations in a particular regulatory region should be present in multiple patients. Given the hundreds of thousands of regulatory elements in the human genome we may be far from having a sample size sufficiently large to detect most functional SNVs. An alternative approach would be to lower the stringency in the statistical pipelines and directly test thousands of “moderate-confidence” SNVs using MPRAs to identify functional variants. Ultimately, a combination of computational and experimental methods along with new technical innovations will increase our ability to identify and characterize the mechanisms by which non-coding SNV drive cancer.

## Author Contributions

KG, SCP, JS, and JFB participated in the writing, reviewing, and critical analysis of the manuscript. JFB prepared the illustrations and coordinated the manuscript. All authors agreed and approved the final version.

## Conflict of Interest Statement

The authors declare that the research was conducted in the absence of any commercial or financial relationships that could be construed as a potential conflict of interest.
